# Does Dietary Vitamin E Improve Efficiency of Selenium Nanoparticles on Reproductive Performance in Marine Fish? Using Arabian Yellowfin Seabream (*Acanthopagrus arabicus*) Female Brooder as an Experimental Model

**DOI:** 10.1155/anu/5995657

**Published:** 2026-06-14

**Authors:** Sadegh Saffari, Mansour Torfi Mozanzadeh, Omid Khademzadeh

**Affiliations:** ^1^ Sika Abzi Jonoub Company, Ahwaz, Khuzestan, Iran; ^2^ South Iran Aquaculture Research Institute, Iranian Fisheries Science Institute (IFSRI), Agricultural Research Education and Extension Organization (AREEO), Ahwaz, Iran, areo.ir; ^3^ Department of Fisheries, Iranian Fisheries Organization, Abadan, Khuzestan, Iran

**Keywords:** α-tocopherol, glutathione peroxidase, nanotechnology, reproduction, seabream

## Abstract

A 90‐day research was carried out to examine the synergistic effects of dietary selenium nanoparticles (SeNs) and vitamin E (Vit E) on the reproduction of female Arabian yellowfin seabream (*Acanthopagrus arabicus*) female brooders (224.9 ± 8.7 g). Seven isoproteic (*~*500 g/kg) and isolipidic (~150 g/kg) plant protein‐rich (60% of fishmeal [FM] replaced with plant protein sources [PPSs]) diets were formulated as follows: (1) control (without SeN and Vit E supplementation), (2) Se^+1^ (supplemented with 1 mg/kg SeN), (3) Se^+1^E^+100^ (supplemented with 1 mg/kg SeN + 100 mg/kg Vit E), (4) Se^+1^E^+250^ (supplemented with 1 mg/kg SeN + 250 mg/kg Vit E), (5) Se^+2^ (supplemented with 2 mg/kg SeN), (6) Se^+2^E^+100^ (supplemented with 2 mg/kg SeN + 100 mg/kg Vit E), and (7) Se^+2^E^+250^ (supplemented with 2 mg/kg SeN + 250 mg/kg Vit E). Two hundred and ninety‐four brooders were stocked into 21 containers (1.5 m^3^) and subjected to the dietary treatments for 90 days. Disinfected seawater (temperature: 20 ± 1.2°C, salinity: 40.2 ± 0.22 g/L) was used in a flow‐through system. The highest (60.2% ± 3.2%) and lowest (37.57% ± 5.2%) fertilization rate percentages were in Se^+2^E^+250^ and control groups, respectively (*p* < 0.05). The lowest hatching rate was in control (55.0% ± 3.6%) and the highest values were in Se^+2^ (73.0% ± 2.0%), Se^+2^E^+100^ (70.5% ± 2.5%), and Se^+2^E^+250^ (67.1% ± 5.6%) groups. The control group had higher abnormal embryogenesis percentage (13.8% ± 1.9%) than the other groups. Selenium concentration in the liver, ovaries, eggs and 3 days post hatch (3DPH) larvae of fish fed Se^+2^, Se^+2^E^+100^ and Se^+2^E^+250^ was higher than the other treatments. Total antioxidant capacity (TAC) and glutathione peroxidase (GPx) activity increased in the liver and ovaries of brooders fed diets supplemented with SeN or in combination with Vit E. Interestingly, TAC significantly increased in the 3DPH larvae by increasing GPx activity, meanwhile catalase (CAT) and superoxide dismutase (SOD) activities and malondialdehyde (MDA) level decreased (*p* < 0.05). Based on these findings, supplementing plant protein‐rich diets with SeN at 2 mg/kg or in combination with 100–250 mg/kg Vit E recommended to promote reproductive performance and antioxidant capacity in *A. arabicus* female brooders.

## 1. Introduction

Fishmeal (FM) has long been a cornerstone of aquafeeds, highly valued for its superior palatability, balanced amino acid profile, excellent digestibility, and high bioavailability of minerals and long‐chain polyunsaturated fatty acids (LC‐PUFA) in its residual fat. However, the escalating demand from a rapidly expanding aquaculture sector, coupled with dwindling wild fish populations, has made FM increasingly expensive and environmentally unsustainable [1]. Indeed, global aquaculture feed production is projected to rise significantly, from 6.54 × 10^10^ kg in 2020–8.71 × 10^10^ kg by 2025, intensifying the pressure for viable alternative protein sources [2].

This critical need has spurred research into sustainable, cost‐effective plant protein sources (PPSs) as substitutes due to their advantages such as lower cost, greater accessibility, and high protein content with reasonable amino acid profile [2]. However, PPS naturally contain high levels of dietary fiber and various antinutritional factors (ANFs), such as phytic acid, that reduce bioavailability of trace elements [3]. The presence of ANFs in PPS is particularly detrimental to the absorption of essential trace elements, and their bioavailability can also be influenced by their chemical form [4]. This is dramatically illustrated by the disparity in selenium (Se) content. For instance, FM typically provides 1.5–3.1 mg Se/kg, whereas many PPS offer a meager <0.01–0.16 mg Se/kg amounts [5]. Consequently, to meet the nutritional requirements in plant protein‐rich diets, trace elements should be supplemented in precise and sufficient amounts.

Selenium is an essential trace element fundamentally integrated into selenoproteins that orchestrate diverse physiological processes. A prime example is selenocysteine, a pivotal component of glutathione peroxidase (GPx) [6]. This enzyme acts as a crucial cellular chaperon, defending biological membranes from oxidative stress. Specifically, GPx neutralizes harmful reactive oxygen species (ROS) like hydrogen peroxide and fatty acid hydroperoxides, converting them into benign water and fatty acid alcohols by utilizing reduced glutathione [6]. Fish dietary Se requirements, typically ranging from 0.15 to 0.70 mg/kg of feed, are dynamic and depend on the species, life stage, and the specific form’s bioavailability [7]. In aquaculture, organic selenium forms generally demonstrate superior bioavailability and beneficial effects compared to their inorganic counterparts. Emerging research further highlights selenium nanoparticles (SeNs) in aquafeeds, showcasing their even greater bioavailability and reduced toxicity relative to both traditional organic and inorganic compounds [7]. These characteristics of SeN enhanced cellular permeability owing to their large surface area and improved functionality through gradual Se release for more efficient absorption [7]. Furthermore, compared to inorganic and organic forms, Se‐N has some privileges, has higher chemical stability and biocompatibility, more water solubility, and better functionality because of the gradual release of Se that leads to its better absorption [7]. Moreover, it has been confirmed that compared to an inorganic source, SeN resulted in greater selenium concentrations in fish tissues [8]. In this context, it has been reported that supplementing diet with SeN resulted in higher selenium deposition in the liver compared to ovaries and progenies in female fish brooders [9–11]. This phenomenon mainly correlated with the upregulation of selenoprotein gene expression in the liver, indicating the vital role of the liver in synthesizing and exporting Se‐containing proteins, facilitating the transport of Se to peripheral tissues, including the gonads [12].

Optimal Se levels are vital for fish brooder reproductive success, as deficiency can prove detrimental [9]. Sufficient dietary SeN boosts antioxidant capacity by activating antioxidant enzymes within gonads, gametes, fertilized eggs, and larvae. This action directly contributes to increase the fertilization rate, hatchability, and larval survivability [9, 10]. Moreover, dietary Se significantly influences the expression of antioxidant‐related genes, further enhancing both antioxidant capacity and reproductive performance in brooders, ultimately leading to higher‐quality offspring [11, 12]. A compelling demonstration of SeN efficacy comes from Naiel et al. [13]. Their study on red tilapia showed that supplementing diets with just 1 mg/kg of SeN compared to sodium selenite or no Se supplementation significantly improved reproductive outcomes. Benefits included larger gonads, earlier and more frequent spawning, extended fertility, enhanced egg biometric indices, and a complete absence of egg deformities. They confirmed that superior reproductive performance in female brooders fed SeN‐supplemented diet compared to sodium selenite groups was mostly related to higher blood luteinizing hormone, follicle‐stimulating hormone, progesterone, and estradiol levels that resulted in a well‐developed stroma structure, a high number of mature vitellogenic oocytes, a remarkable number of well‐developed postvitellogenic oocytes, and no empty follicles in ovaries.

Along with Se, vitamin E (Vit E), a potent family of fat‐soluble compounds encompassing tocopherols and tocotrienols, is a vital structural and functional element in biological systems. As an essential component of cellular membranes, it acts as a primary antioxidant, effectively neutralizing harmful free radicals, such as lipid peroxyl radicals, and thereby safeguarding overall cellular and tissue integrity [14]. Its role in reproductive health is multifaceted and critical, and it has a pivotal role as an endocrine regulator. It enhances gonad development by elevating gonadal steroid hormones while also stimulating the secretion of sex hormones from the anterior pituitary [14]. Crucially, its antioxidant capacity shields fragile sperm cells from debilitating oxidative damage, significantly boosting overall fertility [15]. Practical research confirms these benefits, demonstrating that optimizing dietary Vit E levels markedly increases spawning rates in species such as gilthead seabream (*Sparus aurata*) [16].

The Arabian yellowfin seabream (*A. arabicus*), a commercially significant carnivorous species within the Sparidae family, is a potential candidate for developing marine cage culture in the Persian Gulf and Oman sea region [17]. Its widespread popularity stems from a robust suite of characteristics, such as successful captive reproduction, high fecundity, impressive larval survival rates, streamlined larviculture, satisfactory growth rate, and strong resilience across varying environmental conditions [18, 19]. Recent studies have underscored the importance of dietary micronutrients in maximizing reproductive performance of this species. For instance, supplementing plant protein‐rich diets with 2–4 mg/kg SeN significantly improve reproductive performance in females [9, 10] and boosted sperm quality and fertility in males [20]. Even more specifically, administering diet with SeN to broodstock has enhanced larval quality by successfully modulating thyroid hormones and accelerating metamorphosis [21]. In addition, dietary supplementation with 250 mg/kg of Vit E increased critical reproductive performance that coinciding with enhanced antioxidant capacity in the male and female gonads [19]. Previous studies in Nile tilapia (*Oreochromis niluticus*) juveniles confirmed that SeN alone [22] or in combination with other vitamins such as Vit E [23] or C [24] not only increased growth performance and feed utilization but also enhanced antioxidant status, immunocompetence, and disease resistance in this species. Given these substantial independent benefits, the present study was conducted to evaluate the interactive effects of dietary SeN and Vit E supplementation on the reproductive performance of the *A. arabicus* female brooders by focusing entirely on the oxidative state.

## 2. Materials and Methods

### 2.1. Ethics Statement

The care and treatment of animals complied with the principles and guidelines of ARRIVE (https://arriveguidelines.org/). All experiments and samplings were done based on the ethical recommendations in the guide for the care, protection, and use of laboratory animals approved by the Institutional Animal Care [25].

### 2.2. Experimental Design

The study was initiated in mid‐November by collecting 1–2‐year‐old *A. arabicus* brooders via hook‐and‐line from Khormusi, Khuzestan, Iran. Postcapture, the fish received a 1‐h freshwater bath and then entered a 2‐week quarantine period. The brooders were housed in three 10 m^3^ concrete tanks at a density of 10 fish/m^3^, supplied by a continuous flow‐through system of filtered, disinfected seawater (5 L/min). Minced fish were fed during quarantine to promote acclimatization. Following this, a month‐long adaptation period commenced, during which the fish were fed a commercial pellet diet (Faradaneh, Shahrekord, Iran, 420 g/kg crude protein, 150 g/kg crude fat, 140 g/kg ash, and 100 g/kg moisture). Brooder sex was ascertained through distinct methods: gentle abdominal pressure revealed milt in males, while catheterization was employed for females. Males were characterized by the emission of fluid milt. Female maturation was gauged by oocyte diameter, with individuals selected at stages III (350–400 µm) and IV (400–500 µm). Biometric data were collected from 294 brooders, which were then distributed into 21 polyethylene tanks, each with a 1.5 m^3^ capacity. A deliberate stocking density of 14 fish per tank and a balanced 1:1 sex ratio were maintained. The tanks were filled with 1200 L of seawater that had been disinfected using 20 g/m^3^ chlorine, neutralized with aeration and 10 g/m^3^ sodium thiosulfate, and subjected to a complete daily water exchange via a flow‐through system (*~*1 L/min).

In this study, 60% of FM was replaced by PPS, including soybean, wheat gluten, and corn gluten meals to formulate a basal diet according to Saffari et al. [9]. Experimental diets were designed by incorporating Vit E (100 and 250 mg/kg) and SeN (1 and 2 mg/kg diets) into the basal diet (Table 1). The Vit E (DL‐α‐tocopherol acetate, Sigma, Germany) dosage selection was according to Najafabadi et al. [19], and SeN concentration selection was based on prior studies that evaluated SeN (30–45 nm particle size, CAS number: 7782–49‐2; purity: 99.95%; size: 30–45 nm, 99.95% purity, Iranian Nanomaterial Pioneers Company, Mashhad, Iran) supplementation in *A. arabicus* brooders [9]. Seven isoproteic (*~*500 g/kg) and isolipidic (~150 g/kg) diets were formulated as follows: (1) control (without SeN and Vit E supplementation), (2) Se^+1^ (supplemented with 1 mg/kg SeN), (3) Se^+1^E^+100^ (supplemented with 1 mg/kg SeN + 100 mg/kg Vit E), (4) Se^+1^E^+250^ (supplemented with 1 mg/kg SeN + 250 mg/kg Vit E), (5) Se^+2^ (supplemented with 2 mg/kg SeN), (6) Se^+2^E^+100^ (supplemented with 2 mg/kg SeN + 100 mg/kg Vit E), and (7) Se^+2^E^+250^ (supplemented with 2 mg/kg SeN + 250 mg/kg Vit E). Preparation of the experimental diets commenced by homogenizing the dry ingredients for 20 min. Following this, the calculated doses of Vit E were thoroughly blended into the oils. SeNs, dissolved in distilled water, were then integrated into this base mixture until a uniform, soft dough formed. To create standardized 6 mm pellets, the dough was pelleted through a meat grinder fitted with a stainless‐steel mesh. The resulting pellets were fan‐dried at room temperature for 24 h and subsequently preserved in a freezer (−20°C). The final diets were analyzed for actual Se and Vit E content postpreparation and are reported in Table 1.

**Table 1:** Ingredients and composition of experimental diets.

Treatments							
Ingredients (g/kg^−1^)^a^	Control	Se^+1^	Se^+1^E^+100^	Se^+1^E^+250^	Se^+2^	Se^+2^E^+100^	Se^+2^E^+250^
Fish meal	300	300	300	300	300	300	300
Soybean meal	100	100	100	100	100	100	100
Wheat gluten meal	200	200	200	200	200	200	200
Corn gluten meal	200	200	200	200	200	200	200
Beef gelatin	25	25	25	25	25	25	25
Wheat middling	30	30	29.8	29.5	30	29.8	29.5
DL‐α‐tocopherol acetate	—	—	0.2	0.5	—	0.2	0.5
Fish oil	31	31	31	31	31	31	31
Canola oil	31	31	31	31	31	31	31
Soy lecithin	20	20	20	20	20	20	20
DL‐methionine	3	3	3	3	3	3	3
L‐lysine	5	5	5	5	5	5	5
Se‐free premix^b^	40	40	40	40	40	40	40
Butyric acid	2.5	2.5	2.5	2.5	2.5	2.5	2.5
Vitamin C	2.5	2.5	2.5	2.5	2.5	2.5	2.5
Di‐calcium phosphate	10	10	10	10	10	10	10
Proximate composition (g/kg)							
Crude protein	502.9	495.9	501.8	498.8	500.8	505.5	495.8
Crude lipid	150.4	148	150.6	150.3	148.7	151.1	148.5
Ash	106	110	107	105	102	112	108
Moisture	64	70	68	66	72	65	69
Selenium (mg/kg)	0.67	1.48	1.39	1.52	2.60	2.55	2.71
Vitamin E (mg/kg)	38	45	128	286	48	131	274

^a^Composition of ingredients [fish meal (620 g kg^−1^ crude protein, 150 g kg^−1^ crude lipid), soybean meal (410 g kg^−1^ crude protein, 42 g kg^−1^ crude lipid), wheat gluten meal (500 g kg^−1^ crude protein, 40 g kg^−1^ crude lipid), corn gluten meal (714 g kg^−1^ crude protein, 41 g kg^−1^ crude lipid), beef gelatin (850 g kg^−1^ crude protein, 42 g kg^−1^ crude lipid), wheat middling (120 g kg^−1^ crude protein, 30 g kg^−1^ crude lipid)].

^b^Included: (as mg kg^−1^ of premix): vitamin A, 50,000 (IU kg^−1^); vitamin D3, 10,000 (IU kg^−1^); vitamin B_1_, 20; vitamin B_2_, 10; vitamin B_6_, 3; vitamin K_3_, 15; nicotinamide, 150; calcium pantothenate, 40; Copper (Cu^++^), 30; iron (Fe^++^), 100; zinc (Zn^++^), 150; manganese (Mn^++^), 200.

In the Northwest Persian Gulf, *A. arabicus* typically spawns between mid‐March and mid‐April, when water temperatures range from 19 to 23°C. To assess the effects of different diets on reproduction, brooders were fed experimental diets starting 60 days before the first expected spawning and continuing throughout the 30‐day spawning period. The diets were administered to satiety twice daily (at 9 AM and 3 PM). Temperature (20 ± 1.2°C), salinity (40.2 ± 0.22 ppt), pH (7.9 ± 0.1), and dissolved oxygen (7.4 ± 0.2 ppm) were evaluated weekly. The photoperiod consisted of an initial regime of 10 h of light and 14 h of darkness, which adjusted to 11 h of light and 13 h of darkness during the spawning period.

### 2.3. Sampling

At the end of the feeding trial, three female fish per tank (triplicate) were selected for blood and tissue sampling. The selected fish were first weighed to determine their final body weights. Blood samples were collected from the caudal vein using a sterile syringe, and the blood was transferred to Eppendorf tubes. After centrifugation (5000 g, 10 min, 4°C), the serum was separated and stored at −80°C for subsequent biochemical and hormone analyses. Following blood collection, the fish were euthanized using 300 mg/L 2‐phenoxyethanol [9]. The liver and ovaries were carefully excised to determine the hepatosomatic index (HSI) and gonadosomatic index (GSI). The liver and ovary tissues were then collected and immediately frozen in liquid nitrogen for subsequent measurements. These tissues were stored at −80°C until processing for Se content determination, antioxidant enzyme assays, and gene expression analysis.

Spawning occurred naturally as water temperatures were at 19°C. To collect released eggs, water inflow was halted at 6 PM, and floating eggs were harvested at 8 AM using a conical net with a mesh size of 300 µm. Fertilization rates were assessed by sampling three sets of 100 floating eggs under a microscope at 40x magnification. This sampling methodology was replicated over 3 days for each treatment group (3 days × 3 tanks × 3 beakers = 27 samples per treatment). The following formulas were employed to calculate spawning and fertilization rates, along with other morphometric parameters [9].

### 2.4. Selenium and Vit E Concentrations

Selenium content in test diets and tissues was determined by inductively coupled plasma mass spectrometry (ICP‐MS). Briefly, tissue samples were digested using concentrated nitric acid and hydrogen peroxide, filtered, and diluted with ultrapure water before the analysis. Controlled heating ensured complete organic material dissolution. The resulting solutions were filtered and diluted with ultrapure water for analysis. Certified Se solutions were used to prepare calibration standards, ensuring accurate and precise measurements. Standard reference materials and replicate were employed to analyses as quality control measures to validate our results.

Extraction of DL‐α‐tocopherol from feed and tissue samples was performed using the protocol established by Buttriss and Diplock [26]. Diets were homogenized with distilled water, and 1 mL of the homogenate was combined with 2 mL of pyrogallol ethanol in screw‐cap test tubes. After preparing this mixture, 300 μL of a 50% potassium hydroxide solution was added, and the samples were saponified in a water bath set at 70°C for 30 min. Following saponification, 4 mL of hexane containing butylated hydroxytoluene (BHT) at a 0.05% concentration was added during the cooling process. The contents of the tubes were then shaken vigorously for 1 min. The amount of Vit E in the extracted solution was quantified using high‐performance liquid chromatography (HPLC), employing a Sil Zorbax column and a spectrofluorometric detector. Standard DL‐α‐tocopherol was used as a reference for calculating the values of Vit E in the samples.

### 2.5. Antioxidant Parameters

Samples of the liver, ovaries, eggs, and larvae were defrosted, weighed, and then homogenized in a ratio of 1–9 (w/v) of cold potassium phosphate buffer (0.1 M, pH = 7:4, 4°C) at 10,000 rpm for 60 s close to ice. The homogenate was centrifuged (9000 rpm, 30 min, 4°C); the supernatant was removed and aliquoted (5 × 500 μL) and kept at −80°C. The catalase (CAT) [27], superoxide dismutase (SOD) [28], GPx [29], total antioxidant capacity (TAC) [30], and malondialdehyde (MDA) [31] values were determined by standard methods. The total protein content of the serum or tissue homogenates was determined by the Bradford [32] method.

### 2.6. Statistics

Data were analyzed by applying SPSS software Version 23.0. Kolmogorov–Smirnov and Leven tests were applied for the evaluation of normality and homogeneity of variance, respectively. A two‐way ANOVA was used to evaluate the individual and interactive effects of dietary Se‐NPs and Vit E on physiological responses at *p*  < 0 : 05 for all statistical tests. If effects of independent factors were significant, then these effects were evaluated separately by a one‐way ANOVA followed by Tukey’s comparison of means. In all cases, *p*  < 0 : 05 was considered significant.

## 3. Results

### 3.1. Growth Parameters

There was no mortality during the husbandry trial (Table 2). Growth parameters including final weight and condition factor did not change among groups (*p* > 0.05). Brooders in control and Se^+1^ groups had higher HSI values than the other treatments. Brooders in Se^+2^E^+100^ had the greatest GSI value, and those in control, Se^+1^E^+100^, Se^+1^E^+100^, and Se^+2^E^+250^ had the lowest values. The VSI value in Se^+1^ and Se^+2^ groups was higher than Se^+2^E^+100^ group. The HSI value decreased by supplementing diet with Vit E and influenced by interactive effects of SeN and Vit E (Table 3). The individual and combined effects of dietary SeN and Vit E affected the GSI value. Dietary Vit E at 100 mg/kg significantly reduced VSI value in female brooders (Table 3).

**Table 2:** Somatic indices of *A. arabicus* females fed different diets (Mean ± SE, *n* = 3).

Treatments							
Parameters	Control	Se^+1^	Se^+1^E^+100^	Se^+1^E^+250^	Se^+2^	Se^+2^E^+100^	Se^+2^E^+250^
BW_i_ (g)	218.5 ± 10.5	213.0 ± 20.2	216.0 ± 6.9	226.0 ± 9.2	237.0 ± 26	235.0 ± 15.9	229.0 ± 2.7
BW_f_ (g)	280.5 ± 18.5	287.3 ± 11.6	289.7 ± 25.4	291.7 ± 19.2	293.3 ± 37.0	281.0 ± 44.8	310.7 ± 47.6
Survival (%)^1^	100 ± 0.0	100 ± 0.0	100 ± 0.0	100 ± 0.0	100 ± 0.0	100 ± 0.0	100 ± 0.0
HSI (%)^2^	2.8 ± 0.2^a^	2.9 ± 0.2^a^	1.5 ± 0.2^c^	1.7 ± 0.0^c^	1.8 ± 0.1^c^	1.7 ± 0.1^c^	2.2 ± 0.3^b^
GSI (%)^3^	2.5 ± 0.4^c^	4.5 ± 0.3^b^	2.4 ± 0.2^c^	2.5 ± 0.9^c^	4.1 ± 0.1^b^	6.8 ± 0.2^a^	2.4 ± 0.9^c^
VSI (%)^4^	2.7 ± 0.1^ab^	3.6 ± 0.4^a^	3.2 ± 0.2^ab^	2.7 ± 0.4^ab^	3.8 ± 0.1^a^	2.2 ± 0.5^b^	2.8 ± 0.1^ab^
K (%)^5^	2.0 ± 0.0	2.1 ± 0.1	2.0 ± 0.0	2.0 ± 0.1	2.0 ± 0.1	2.1 ± 0.2	1.9 ± 0.4

*Note:* Means in the same row with different superscripts are significantly different (*p*  < 0.05). *K*, Fulton’s condition factor. BWi, initial body weight; BWf, final body weight.

Abbreviations: GSI, gonadosomatic index; HSI, hepatosomatic index; VSI, viscerosomatic index.

^1^Survival rate (%): (number of fish remaining on day 90/initial number of fish) × 100.

^2^Hepatosomatic index (HSI %): (liver weight [g]/final weight [g]) × 100.

^3^Gonadosomatic index (GSI %): (gonad weight [g]/final weight [g]) × 100.

^4^Viscerosomatic index (VSI %): (visceral weight [g]/final weight [g]) × 100.

^5^Fulton’s condition index (K): (final weight [g]/standard length^3^ [cm]) × 100.

**Table 3:** Two Way ANOVA analysis evaluated the independent and interaction effects of dietary SeN and Vit E on physiological responses of *A. arabicus* females (*p* < 0.05).

Parameters	SeN	Vit E	Interaction
BW_i_	0.888	0.654	0.987
BW_f_	0.507	0.964	0.586
Survival	1.000	1.000	1.000
HSI	0.957	**0.002**	**0.002**
GSI	**0.015**	**0.005**	**0.002**
VSI	0.457	**0.047**	0.337
K	0.958	0.888	0.954
RF	**0.001**	**0.001**	**0.001**
Spawning period	**0.004**	0.178	0.178
Spawning per day	**0.047**	**0.008**	0.072
Number of egg/mL	**0.001**	**0.001**	**0.001**
Number of egg/g	**0.001**	**0.001**	**0.001**
Egg diameter	**0.001**	**0.001**	**0.009**
Egg’s oil glob diameter	**0.003**	**0.001**	0.329
Buoyant eggs	**0.004**	0.333	**0.061**
Fertilization rate	**0.001**	**0.001**	**0.001**
Hatching rate	**0.001**	**0.001**	**0.001**
Abnormal embryogenesis	0.158	0.971	**0.001**
Larvae length	**0.001**	**0.001**	0.713
Larvae yolk length	**0.016**	0.252	0.777
Larvae oil glob diameter	**0.042**	0.230	0.777
3DPH larvae length	**0.001**	0.221	0.762
3DPH larvae oil glob diameter	0.373	0.749	0.142
Se concentration in liver	**0.001**	**0.041**	0.332
Se concentration in ovary	**0.001**	0.162	0.915
Se concentration in egg	**0.001**	0.238	0.231
Se concentration in 3DPH larvae	**0.001**	0.475	0.426
CAT activity in liver	**0.046**	0.515	0.110
CAT activity in ovary	0.235	**0.019**	0.574
CAT activity in egg	0.328	0.467	0.190
CAT activity in 3DPH larvae	**0.024**	0.185	0.964
SOD activity in liver	0.057	0.238	0.760
SOD activity in ovary	0.175	0.135	0.954
SOD activity in egg	0.145	0.328	**0.036**
SOD activity in 3DPH larvae	0.326	0.718	**0.045**
GPx activity in liver	**0.038**	0.302	0.694
GPx activity in ovary	**0.003**	0.599	0.656
GPx activity in egg	**0.002**	**0.004**	**0.008**
GPx activity in 3DPH larvae	**0.009**	**0.004**	0.643
TAC activity in liver	**0.008**	0.339	0.984
TAC activity in ovary	**0.028**	0.687	0.964
TAC activity in egg	**0.001**	0.822	0.879
TAC activity in 3DPH larvae	**0.001**	0.339	0.964
MDA level in liver	**0.017**	0.107	0.315
MDA level in ovary	**0.001**	**0.001**	0.577
MDA level in egg	0.200	0.939	**0.005**
MDA level in 3DPH larvae	0.688	0.148	**0.021**

*Note:* Values in bold are statically significant (*p* < 0.05). *K*, Fulton’s condition factor. BWi, initial body weight; BWf, final body weight.

Abbreviations: CAT, catalase; DPH, daily posthatch; GPx, glutathione peroxidase; GSI, gonadosomatic index; HSI, hepatosomatic index; MDA, malondialdehyde; RF, relative fecundity; SOD, superoxide dismutase; TAC, total antioxidant capacity; VSI, viscerosomatic index.

### 3.2. Reproductive Performance

The highest and lowest relative fecundity values were in Se^+1^ and control groups (*p* < 0.05, Table 4). Brooders in Se^+2^E^+250^ group had the longest spawning period (17 days) compared to the other groups, and it was increased by supplementing diet with SeN (Table 3). Brooders in Se^+1^ had higher spawning per day (43.0 ± 4.4 egg × 10^3^) than the other treatments, and it was decreased by supplementing diet with 2 mg SeN or dietary Vit E inclusion. Number of eggs per mL in Se^+1^ and Se^+1^E^+250^ groups was greater than the others and those in Se^+2^E^+250^ had the lowest value (*p* < 0.05). The greatest and least eggs per g were in Se^+1^E^+250^ and Se^+2^E^+250^, respectively. Egg diameter in Se^+2^ and Se^+2^E^+250^ groups was higher than the other treatments. Egg’s oil glob diameter in Se^+1^E^+250^, Se^+2^, and Se^+2^E^+250^ was higher than other treatments. The highest buoyant egg percentage was in Se^+2^E^+250^ and the lowest ones were in control and Se^+1^E^+250^ (*p* < 0.05). The highest (60.2 % ± 3.2%) and lowest (37.57 % ± 5.2%) fertilization rate percentages were in Se^+2^E^+250^ and control groups, respectively (*p* < 0.05). The lowest hatching rate was in control (55.0% ± 3.6%), and the highest values were in Se^+2^ (73.0% ± 2.0%), Se^+2^E^+100^ (70.5% ± 2.5%), and Se^+2^E^+250^ (67.1% ± 5.6%) groups. The number of eggs per mL or g, egg diameter, fertilization, and hatching rates were significantly affected by individual or combined effects of dietary Vit E and SeN (Table 3). The control group had a higher abnormal embryogenesis percentage (13.8% ± 1.9%) than the other groups. The greatest larval and yolk sac lengths were in Se^+2^ and Se^+2^E^+250^, and the lowest values were in the control group. Oil glob diameter in larvae was greatest in Se^+2^E^+250^, and the lowest values were in the control group. The shortest 3 days post hatch (3DPH) larval length was in the control group, but those in Se^+2^, Se^+2^E^+100^, and Se^+2^E^+250^ had the longest 3DPH larval length. Supplementing diet with 2 mg/kg SeN significantly increased larval length, yolk sac length, oil glob diameter, and 3DPH larval length (Table 3). The oil glob diameter in 3DPH larvae did not change among the treatments.

**Table 4:** Reproductive performance and larvae characteristics of *A. arabicus* females fed different diets (mean ± SE, *n* = 3).

Parameters	Treatments						
	Control	Se^+1^	Se^+1^E^+100^	Se^+1^E^+250^	Se^+2^	Se^+2^E^+100^	Se^+2^ *E* ^+250^
RF (egg/g female)	411.2 ± 27.7^d^	698.4 ± 28.9^a^	528.2 ± 16.2^c^	579.9 ± 11.5^b^	499.9 ± 17.3^c^	483.2 ± 7.5^c^	523.5 ± 4.4^c^
Spawning period (day)	14.0 ± 0.6^b^	14.0 ± 0.6^b^	14.0 ± 0.6^b^	15.0 ± 0.6^b^	15.0 ± 0.6^b^	16.0 ± 0.6^ab^	17.0 ± 1.0^a^
Spawning per day (egg × 10^3^)	38.1 ± 1.9^b^	43.0 ± 4.4^a^	27.1 ± 2.3^c^	31.3 ± 1.7^c^	31.4 ± 2.9^c^	28.5 ± 1.7^c^	28.7 ± 2^c^
Number of egg/mL	1302.0 ± 13.3^c^	1426.5 ± 16.6^a^	1381.5 ± 11.4^b^	1454.5 ± 35.6^a^	1297.8 ± 24.3^c^	1320.8 ± 17.6^c^	1132.0 ± 14.9^d^
Number of egg/g	2395.5 ± 18.8^ab^	2498.8 ± 47.5^ab^	2512.8 ± 30.0^ab^	2583.8 ± 85.9^a^	2400.8 ± 33.4^ab^	2381.5 ± 142.5^ab^	2295.8 ± 32.9^b^
Egg diameter (µm)	777.3 ± 4.3^c^	776.3 ± 3.4^c^	767.5 ± 2.6^c^	787.5 ± 3.4^b^	803.8 ± 3.3^a^	776.3 ± 3.8^c^	812.5 ± 2.8^a^
Egg’s oil glob diameter (µm)	184.6 ± 2.4^b^	188.1 ± 2.8^b^	185.0 ± 2.8^b^	192.5 ± 2.5^a^	198.1 ± 2.1^a^	187.5 ± 2.7^b^	198.8 ± 2.0^a^
Buoyant eggs (%)	40.8 ± 3.8^b^	46.7 ± 4.4^ab^	42.2 ± 2.3^ab^	39.5 ± 5.4^b^	49.9 ± 3.6^ab^	46.4 ± 1.2^ab^	55.2 ± 2.9^a^
Fertilization rate (%)	37.57 ± 5.2^c^	44.6 ± 3.6^b^	40.8 ± 3.9^b^	43.2 ± 3.2^b^	52.2 ± 4.9^ab^	56.4 ± 3.7^ab^	60.2 ± 3.2^a^
Hatching rate (%)	55.0 ± 3.6^c^	60.8 ± 3.3^b^	60.8 ± 6.0^b^	60.7 ± 3.9^b^	73.0 ± 2.0^a^	70.5 ± 2.5^a^	67.1 ± 5.6^a^
Abnormal embryogenesis (%)	13.8 ± 1.9^a^	6.9 ± 0.7^b^	7.4 ± 0.7^b^	7.0 ± 0.9^b^	6.4 ± 0.6^b^	6.1 ± 0.7^b^	6.2 ± 0.7^b^
Larvae length (µm)	1681.3 ± 11.2^c^	1751.3 ± 9.2^b^	1718.8 ± 13.6^bc^	1752.5 ± 10.6^b^	1797.5 ± 13.3^a^	1756.3 ± 12.4^b^	1808.0 ± 8.2^a^
Larvae yolk length (µm)	529.2 ± 10.3^c^	602.5 ± 6.0^ab^	578.8 ± 5.8^b^	597.5 ± 7.6^ab^	620.0 ± 12.1^a^	608.8 ± 16.4^ab^	613.8 ± 12.0^a^
Larvae oil glob diameter (µm)	165.6 ± 3.3^c^	175.0 ± 3.6^b^	180.0 ± 4.3^ab^	178.8 ± 3.8^ab^	180.0 ± 4.3^ab^	183.8 ± 3.3^ab^	188.8 ± 2.9^a^
Three DPH larvae length (µm)	2520.6 ± 27.6^c^	2654.7 ± 23.6^b^	2631.6 ± 23.5^b^	2641.4 ± 24.2^b^	2749.7 ± 20.3^a^	2694.7 ± 20.8^a^	2711.4 ± 23.6^a^
Three DPH larvae oil glob diameter (µm)	75.0 ± 4.1	72.5 ± 1.7	72.5 ± 2.8	70.6 ± 2.4	70.0 ± 2.5	73.1 ± 3.4	78.1 ± 2.2

Note: Means in the same row with different superscripts are significantly different (*p* < 0.05). Relative fecundity: total number of produced eggs/fish weight (g). Fertilization rate: total number of cleaving oocytes/total number of incubated oocytes × 100.

Abbreviations: DPH, daily posthatch; RF, relative fecundity.

### 3.3. Se Concentration

**Figure 1:**
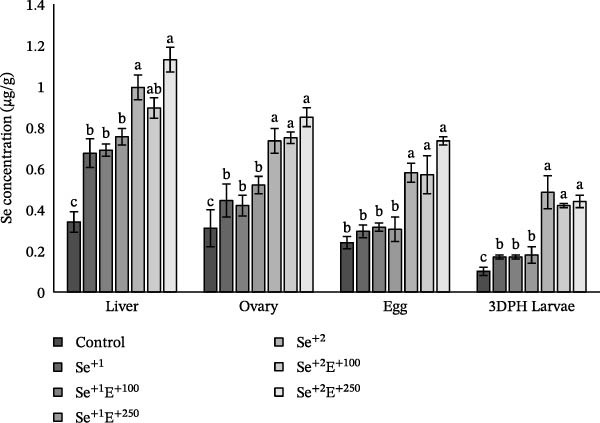
Selenium concentration (µg/g wet weight of tissue) in the liver, ovary, eggs, and 3 days post hatch larvae of *A. arabicus* female brooders fed the experimental diets. Data are expressed as mean ± SE, and diverse little letters show significant differences in different groups (*p* < 0.05).

Selenium concentration in the liver, ovaries, eggs, and 3DPH larvae of fish fed Se^+2^, Se^+2^E^+100^, and Se^+2^E^+250^ was higher than the other treatments and control group had the lowest Se levels in these tissues (Figure 1, *p*  < 0.05). Dietary 2 mg SeN and 250 mg Vit E significantly increased Se retention in the liver; however, in the other tissues, Se concentration increased by supplementing diet with 2 mg SeN (Table 3).

### 3.4. Antioxidant Factors

**Figure 2:**
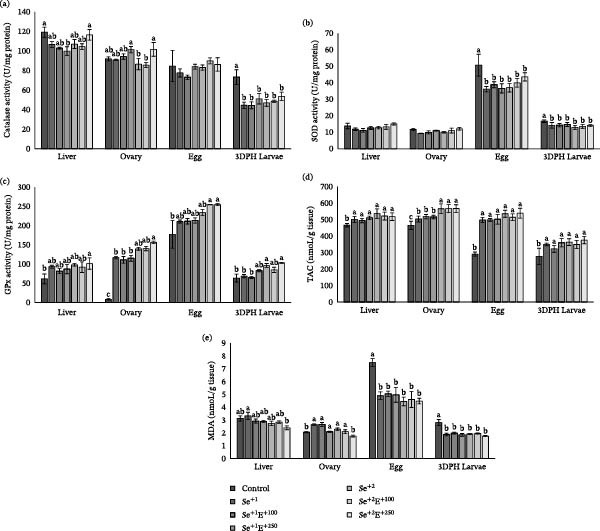
(a) Catalase (CAT), (b) superoxide dismutase (SOD), (c) glutathione peroxidase (GPx), (d) total antioxidant capacity (TAC), and (e) malondialdehyde (MDA) concentration in the liver, ovary, eggs, and 3 days post hatch larvae in female Arabian yellowfin sea bream (*Acanthopagrus arabicus*) fed the experimental diets. Data are mean ± SD (*n* = 9). For each parameter, values with different letters show significant differences (*p*  < 0.05). Parameters with no superscripts show no significant differences among groups (*p* > 0.05).

CAT activity in the liver of control and Se^+2^E^+250^ groups was higher than Se^+1^E^+250^ (Figure 2a, *p*  < 0.05) and it was decreased by supplementing diet with 1.0 mg SeN (Table 3). In the ovaries, brooders in Se^+1^E^+250^ and Se^+2^E^+250^ had higher CAT activities than Se^+2^ and Se^+2^E^+100^, and it was reduced by supplementing diet with 100 mg/kg Vit E. In the eggs, CAT activity did not change among the experimental groups. In 3DPH larvae, CAT activity in the control group was significantly higher than in other treatments (*p* < 0.05).

SOD activity did not change in the liver and ovary tissues (Figure 2b, *p*  < 0.05). The amount of SOD activities in the eggs and 3DPH larvae in the control was higher than the other groups, and it was affected by the combined effects of dietary Vit E and SeN (Table 3).

The highest and lowest GPx activities in the liver and ovaries were in Se^+2^E^+250^ and control groups, respectively (Figure 2c), and it was increased by supplementing diet with 2 mg/kg SeN (Table 3). GPx activity in the eggs of brooder fed Se^+2^E^+100^ and Se^+2^E^+250^ was higher than control, and it was increased by supplementing diet 2 mg/kg SeN and Vit E supplementation. In 3DPH larvae, GPx activity in Se^+2^ and Se^+2^E^+250^ was higher than control, Se^+1^, and Se^+1^E^+100^ groups, and it was affected by individual and combined effects of dietary SeN and Vit E.

TAC (Figure 2d) in the control group was lower than other experimental groups and supplementing diet with 2 mg/kg SeN markedly increased TAC level irrespective of dietary Vit E level in diet (Table 3).

The highest and lowest MDA levels in the liver were in Se^+1^ and Se^+2^E^+250^, respectively (Figure 2e, *p*  < 0.05). The amount of MDA in the ovaries of the control and Se^+2^E^+250^ groups was lower than in the other treatments. The MDA levels in the liver and ovaries significantly decreased by supplementing the diet with 2 mg/kg SeN (Table 3). The values of MDA in the eggs and 3DPH larvae of control were higher than the other treatments, and it was affected by interactive effects of dietary SeN and Vit E (*p* < 0.05).

## 4. Discussion

### 4.1. Growth Parameters and Se Deposition

Supplementation of the PP‐rich diet with SeN, Vit E, or their combination yielded no significant effect on the growth or somatic indices of *A. arabicus*. This finding suggests that the baseline selenium (0.67 mg/kg) or Vit E (38 mg/Kg) concentrations in the basal diet were probably sufficient to satisfy the species’ basic physiological and reproductive requirements [9]. The observed lack of somatic response is primarily attributed to biological energy partitioning during the reproduction cycle. During the spawning season, organisms prioritize gonadal maturation, including gametogenesis and gonadal development. Consequently, the substantial portion of dietary nutrients and metabolic energy is preferentially channeled toward reproductive processes, which intrinsically reduces expenditure on body mass [17]. Previous studies showed that supplementing the diet with various levels of SeN [9] or Vit E [19] did not affect growth parameters in *A. arabicus* brooders. Also, neither dietary Se supplementation for rainbow trout (*Oncorhynchus mykiss*) [11] nor Vit E for *O. niloticus* [33] had a discernible impact on the growth of their respective broodstock.

The HSI in female breeders is a critical measure that reflects the liver pivotal metabolic role in reproduction, specifically the mandatory synthesis of vitellogenin and other yolk precursors during vitellogenesis [34]. In this study, HSI values in control and Se^+1^ groups were higher than brooders fed Se^+2^ or combination of Vit E and Se. In this regard, Saffari et al. [9] reported dietary SeN at 2 mg/kg increased HSI in *A. arabicus* female brooders; meanwhile, Khorasaninasab et al. [35] showed supplementing the diet with 2 mg/kg SeN reduced HSI in Asian seabass (*L. calcarifer*) female brooders. The lower HSI observed specifically in the brooders fed a combination of SeN and Vit E suggests that the supplements did not increase liver mass but instead optimized hepatic function. In this context, it has been proved that sufficient amount of Se [36] or Vit E [37] levels are known to bolster liver function by drastically improving antioxidant defenses. By reducing the high oxidative demands placed on the hepatic tissue during reproduction, Se and Vit E supplementation could lower the overall metabolic workload required for vitellogenesis. Consequently, the reduced HSI indicates that reproductive nutritional needs were met through enhanced functional efficiency rather than through a compensatory increase in relative liver size [35]. Our study revealed significantly elevated GSI values in female breeders receiving the Se^+2^E^+100^‐supplemented diet compared to the other groups. This outcome suggests the combination of SeN and Vit E at 2 and 100 mg/kg, respectively, promotes resource allocation towards reproductive growth, likely by supporting the synthesis of sex steroids and gametogenesis [19, 29]. In this regard, supplementing diet with SeN (2 mg/kg) or a combination of SeN and Vit E (1 mg + 100 mg/kg) increased GSI in *L. calcarifer* [35] and *O. niloticus* [33] female brooders, respectively.

Dietary supplementation with SeN resulted in marked Se bioaccumulation in the liver and ovaries of female *A. arabicus* brooders. Crucially, this reproductive deposition translated into successful maternal transfer, evidenced by significantly elevated eggs and whole‐body Se content in 3‐day‐old larvae in fish fed SeN solely or in combination with Vit E compared to the control. This transfer mechanism is vital for delivering essential trace elements to developing embryos, thereby fortifying their antioxidant defenses during vulnerable early life stages [35]. Interestingly, the combination of SeN and Vit E increased Se retention in the liver of *A. arabicus* female brooders, suggesting that Vit E has a synergistic effect on selenoproteins synthesis and accumulation in this organ. Observations in other fish species confirm this pattern: Se is concentrated in maternal reservoirs (liver and ovaries) and then mobilized into the eggs to support development and confer protection against oxidative stress [10, 11, 35]. This mobilization relies on the liver’s synthesis of vitellogenin, an essential yolk precursor protein that binds and transports necessary micronutrients, including Se, to the developing oocytes.

### 4.2. Reproductive Performance

Selenium deficiency is a well‐established factor limiting reproductive performance in various fish species that results in offspring survival reduction [38, 39]. In addition, Vit E is indispensable for reproductive health, functioning as a key component in the synthesis of sex hormones and promoting gonadal development and gamete quality, such as enhancing yolk granule formation in animal ovaries [40, 41]. Specifically, Vit E is credited with upregulating key circulating gonadotropin hormones, which leads to practical benefits such as higher larval numbers, increased size of healthy eggs, and accelerated spawning time [41]. This powerful effect is because of its mobilization mechanism: Vit E stores accumulated in different fish organs are efficiently transferred to the gonads just before spawning [41]. This supply is not only vital for spawning performance but is also crucial for subsequent embryonic development [42].

Based on this, the results of the present study demonstrated that dietary enrichment with 2 mg/kg SeN and 250 mg/kg Vit E significantly boosted the reproductive success of brooders by increasing spawning period, egg diameter, egg’s oil droplet diameter, buoyant eggs, fertilization and hatching rates percentage, and enhanced larval length and survival in *A. arabicus* female brooders. This highlights a clear synergistic relationship between Se and Vit E supplementation on overall fish reproductive outcomes. The results, specifically the significantly higher fertilization and hatching rates and the marked decrease in abnormal embryogenesis observed fish fed diet supplemented with SeN and Vit E compared to control, provide compelling evidence that these supplements substantially enhanced reproductive success in *A. arabicus* brooders. By bolstering the antioxidant defense, SeN and Vit E maximize gametes functionality, fertilizing potential, and hatching rate [9, 19].

Crucially, the reduction in developmental abnormalities suggests that the protective role of Se and Vit E extends into the early embryonic stage. Since the rapid cellular division of early embryogenesis is highly susceptible to oxidative stress, a known impediment to viability and normal differentiation [9], the SeN and Vit E supplementation likely alleviated this stressor, resulting in more robust developmental outcomes. This finding aligns with previous studies demonstrating that paternal SeN and Vit E supplementation effectively improves offspring quality by mitigating oxidative damage in both gametes and nascent embryos [7, 19]. In this regard, Ghanem et al. [43] reported that supplementing diet with 1 mg SeN and 100 mg/kg Vit E increased egg’s diameter, fry weight, and survival rate in *O. niloticus* that was associated with increased plasma sexual hormones and gonad development in this species. Moreover, Saffari et al. [9] reported that supplementing diet with 2–4 mg/kg SeN increased egg size, buoyant egg, fertilization, and hatching rates and markedly boosted larval survival rate and length in *A. arabicus* female brooder. Also, Wischhusen et al. [11] and Khorasaninasab et al. [35] similarly reported that Se supplementation increased the number of spawning and improved offspring survival in *O. mykiss* and *L. calcarifer*, respectively. In addition, supplementing the diet of female *A. arabicus* with 250 mg of Vit E significantly boosted their reproductive output, leading to greater fecundity, longer spawning duration, higher fertilization and hatching rates, fewer embryonic abnormalities, and improved larval survival [19]. Similarly, Erdogan and Arslan [44] found that supplementing the diet of the cichlid species *Pseudotropheus socolofi* with 219.3 mg/kg of Vit E significantly enhanced several reproductive outcomes. These improvements included increased egg diameter, greater fecundity, higher spawning frequency, and improved fertilization rates, alongside better hatching success and larval survival.

### 4.3. Antioxidant Capacity

Regardless of its dietary form, selenium (Se) is ultimately converted to selenocysteine (Sec) and incorporated into crucial antioxidant selenoproteins. This incorporation relies on a specialized mechanism that decodes the UGA codon in mRNA, guided by the selenocysteine insertion sequence [45]. Moreover, Vit E plays a pivotal role in cellular membrane preservation, acting as a powerful antioxidant that underpins a range of biological processes [14]. In fish, this is particularly crucial. Their diets are abundant in LC‐PUFA, which readily undergo oxidation. Vit E antioxidant properties are essential for shielding these susceptible fats from oxidative damage [14].

Central to the antioxidant system in aerobic organisms are enzymes like GPx, SOD, and CAT, which are essential for neutralizing ROS [46]. CAT, a metalloprotein oxidoreductase, plays a vital role in maintaining the redox balance and regulating hydrogen peroxide, thus protecting against oxidative stress [47]. The results of the present study showed that CAT activity responded differently in various tissues. For instance, CAT activity relatively increased in the liver of fish fed control and Se^+2^E^+250^, indicating an increased requirement for hydrogen peroxide scavenging. On the other hand, higher CAT activity in the ovaries of brooders fed Se^+1^E^+250^ and Se^+2^E^+250^ might be related to a robust compensatory mechanism. This response actively works to counteract ROS accumulation, demonstrating the dynamic regulation of the tissue’s antioxidant defenses. In the 3DPH larvae, CAT activity in groups offered with diets containing Se, Vit E, or their combination decreased, suggesting improved antioxidant status in these groups compared to the control. Since no significant changes were detected in egg’s CAT activity, it is plausible that CAT does not hold a primary role in the antioxidative protection of *A. arabicus* eggs. In a study, Saffari et al. [10] reported that supplementing diet with 0.5–1 mg/kg SeN increased CAT activity in liver, ovaries, and larvae of *A. arabicus*, but higher concentrations (2–4 mg/kg) considerably reduced its activity. In contrast, Khorasaninasab et al. [35] reported that supplementing diet with 2 mg/kg SeN reduced CAT activity in the liver and ovaries of *L. calcarifer*, but it increased in larvae, indicating CAT activity is species‐specific. On the other hand, Najafabadi et al. [19] reported that supplementing diet with 100 mg/kg Vit E increased CAT activity in the liver, ovaries, and larvae of *A. arabicus*, but higher or lower concentrations suppressed its activity.

SODs, which are metalloenzymes, represent the primary line of antioxidant defense by converting superoxide radicals [48]. In the present study, SOD activity in the eggs and 3DPH larvae of groups offered with diets containing SeN, Vit E, or their mixture considerably decreased, indicating promoted antioxidant capacity in these groups, which was confirmed by higher TAC and lower MDA values in these treatments compared to the control. In this context, Saffari et al. [9] reported that supplementing diet with 1.0 mg/kg SeN reduced SOD activity in the liver and ovaries of female *A*. rabicus brooders; meanwhile, at 4 mg/kg decreased SOD activity in 3DPH larvae. In another study, Khorasaninasab et al. [35] reported that supplementing diet with 2 mg/kg SeN did not affect SOD activity in the liver, ovary, and larvae of *L. calcarifer* female brooders. It seems that in our research, the combination of SeN with Vit E in this study promoted their synergistic effects on antioxidant capacity of larvae.

The antioxidant benefits of Se are generally attributed to its role in synthesizing Se‐dependent GPx [6], and GPx activity serves as a reliable indicator of Se bioavailability [49]. Furthermore, it has been reported that GPx activity increased in *L. calcarifer* juveniles fed oxidized fish oil and 2 mg/kg SeN, which correlated with upregulation of GPx gene expression in the liver of this species [50]. The results of the current research showed that GPx activity in the liver, ovaries, and eggs of fish in the control group was lower than that in the other groups, particularly Se^+2^E^+250^, indicating that supplementing the diet with SeN and Vit E boosted GPx activity in *A. arabicus* female brooders. In larvae, Se^+1^E^+250^, Se^+2^, Se^+1^E^+100^, and Se^+2^E^+250^ had relatively higher GPx activity compared to the other groups, suggesting high level of SeN at 2 mg/kg or combination of SeN at 1 mg/kg with a high level of Vit E (250 mg/kg) could induce higher GPx activity at the larval stage. In this regard, Saffari et al. [10] reported that supplementing diet with 1–4 mg/kg SeN increased GPx activity in liver, ovaries, and 3DPH larvae of female *A. arabicus*. In addition, Khorasaninasab et al. [35] reported that supplementing diet with 2 mg/kg SeN increased GPx activity in the ovaries and larvae of *L. calcarifer* female brooders. Moreover, enrichment of livefoods with 5 mg/L enrichment solution increased GPx activity in *A. arabicus* larvae [51]. On the other hand, Najafabadi et al. [19] showed that the inclusion of only 25 mg/kg Vit E increased GPx activity in the liver, ovaries, and larvae of female *A. arabicus*, but higher GPx activity in higher concentrations of Vit E showed fluctuations in various tissues. Thus, based on the above‐mentioned findings, it is recommended to determine the antioxidant potential of an antioxidant nutrient such as Se or Vit E by the presence of other antioxidants in the diet to provide more realistic results.

TAC, encompassing both enzymatic and nonenzymatic components, is a valuable metric for assessing male fertility in teleosts, with its seminal plasma levels varying by species, underscoring inherent physiological differences [52]. In the current study, TAC in the liver, ovaries, eggs, and 3DPH larvae of fish fed diets supplemented with SeN, Vit E, or their combination increased significantly compared to control that associated with the increment of GPx activity. In this regard, Saffari et al. [10] reported that TAC in the liver, ovaries, eggs, and 3DPH larvae of female *A. arabicus* considerably increased by the inclusion of 2–4 mg SeN in diet. In addition, enrichment of livefoods with 1–5 mg/L enrichment solution increased TAC activity in *A. arabicus* larvae [51]. Furthermore, Najafabadi et al. [19] reported that supplementing the diet with 500–1000 mg/kg Vit E increased TAC in the liver and ovaries of *A. arabicus* female brooders; meanwhile, supplementing the diet with 100 mg/kg Vit E boosted TAC in their larvae.

Oxidative damage, particularly lipid peroxidation, can be measured by MDA levels [53]. The liver, being metabolically active, and ovaries enriched with LC‐PUFA that make them highly susceptible to oxidative damage by ROS [9]. In the present research, MDA levels in the liver and ovaries of female brooders were lower than other groups, indicating the highest concentrations of SeN (2 mg/kg) and Vit E (250 mg/kg) could efficiently control lipid peroxidation in these tissues. Furthermore, MDA levels in eggs and 3DPH larvae of brooders fed diets containing SeN, Vit E, or their blends noticeably alleviated that was in concomitant with higher GPx activity and TAC values in these treatments compared to the control. Other studies in the female brooders of *A. arabicus* also confirmed supplementing diet with SeN [10] or Vit E [19] reduced MDA levels in the liver, ovaries, and larval stage. Also, enrichment of livefoods [51] or formulated diet [54] with SeN significantly decreased the MDA level in larval and juvenile stages of *A. arabicus*, respectively. The findings of our study clearly showed that synergistic effects of Vit E and SeN by increasing antioxidant capacity not only curb lipid peroxidation but also improve reproductive performance and larval survivability in *A. arabicus*.

## 5. Conclusion

In summary, the findings of the present research showed that the synergistic effects of dietary SeN and Vit E can improve reproductive performance in female *A. arabicus* brooders, which is mainly attributed to increased Se retention and elevated antioxidant capacity in this species. In this study, supplementing diet solely with SeN at 2 mg/kg or in combination with 100–200 mg/kg Vit E considerably improved reproductive efficiency in *A. arabicus*, particularly in Se^+2^E^+250^ that significantly prolonged spawning duration. In addition, Se retention in the liver, ovaries, egg, and 3DPH larvae markedly increased in fish fed diets supplemented with SeN at 2 mg/kg or in combination with 100–200 mg/kg Vit E. TAC increased in the liver and ovaries of brooders fed diets supplemented with SeN or in combination with Vit E compared to the control group, which was mainly attributed to GPx activity augmentation in these tissues. Interestingly, antioxidant capacity significantly increased in the progeny by increasing GPx activity and TAC values that associated with reduction of CAT and SOD activities and decrease in MDA values. Further studies are required to evaluate key reproductive hormones (e.g., 17 beta‐estradiol and testosterone) and related gene expression data (e.g., vitellogenin and gonadotropin receptors) to elucidate the mode of action of Vit E and SeN and the link between them on reproductive performance of *A. arabicus*.

## Author Contributions

**Sadegh Saffari:** methodology, formal analysis. **Mansour Torfi Mozanzadeh**: writing – original draft, supervision, investigation, conceptualization, methodology, formal analysis. **Omid Khademzadeh**: methodology, formal analysis.

## Funding

No funding was received for this manuscript.

## Disclosure

All authors review and approve the manuscript for publication.

## Consent

The authors have nothing to report.

## Conflicts of Interest

The authors declare no conflicts of interest.

## Data Availability

This data will be made available upon request.
